# Resistant hypertension and PRES syndrome induced by carbamazepine in a patient with SLE: A case report and literature review

**DOI:** 10.1016/j.amsu.2022.103767

**Published:** 2022-05-11

**Authors:** Mohammad Alsultan, Kassem Basha

**Affiliations:** a Department of Nephrology, Al Assad and Al Mouwasat University Hospitals, Damascus University- Faculty of Medicine, Damascus, Syria; b Nephrology Department, Al Mouwasat University Hospitals, Damascus University- Faculty of Medicine, Damascus, Syria

**Keywords:** Resistant hypertension, Posterior reversible encephalopathy syndrome, Carbamazepine, SLE, ESRD

## Abstract

**Introduction:**

Several conditions of resistant hypertension (RHTN) have been suggested and are often associated in the same patient. Approximately 75% of patients with posterior reversible encephalopathy syndrome (PRES) have moderate to severe HTN at presentation.

**Case presentation:**

A 26- year old SLE-patient presented with seizures followed by confusion and cortical blindness, in the context of emergent HTN and MRI revealed PRES syndrome. However, antihypertensive drugs were increased to maximum doses with two HD sessions, the patient still had high measures of BP. The dilemma was to find the underlying cause of long-term RHTN in this patient, where several etiologies were implicated. We review the status in more specific details and draw a timeline, which showed constant exposer to carbamazepine from the beginning of HTN. Thereafter, converting the patient to levetiracetam resulted in resolving the RHTN.

**Discussion/conclusion:**

We discuss this case with a literature review over the past ten years, which shows only three patients with a neurologic deficit in the context of severe HTN induced by carbamazepine. In the end, determining the secondary etiology of RHTN, in this patient, is considered a diagnosis of challenge due to the coincidence with SLE and the rarity of this side effect of carbamazepine. This is considered a valuable message to always exclude all secondary causes, especially drugs effects, in ESRD-patients with multiple comorbidities.

## Highlights

• Carbamazepine caused resistance to almost all hypertensive classes.
• Carbamazepine seems not dose-dependent since it was associated with HTN even at very low doses and low therapeutic levels.
• In our case, RHTN is most likely due to chronic reduction of antihypertensive levels.
• Conversion of carbamazepine to levetiracetam yielded in the resolve of RHTN.
• Side effects of drugs should always exclude in the approach of RHTN especially in ESRD-patients with multiple comorbidities.

## 1 Introduction

Resistant Hypertension (RHTN), affecting 20–30% of the different populations, is defined as high blood pressure (BP) that remains uncontrolled (>140/90 mm Hg) despite the use of effective doses of three or more different classes of antihypertensive agents, including a diuretic. Several factors have been suggested to be causes for resistance; such as noncompliance or inadequate doses or combinations of drugs, volume overload, drug-induced (NSAIDs, sympathomimetics, corticosteroids, erythropoietin), and secondary hypertension (primary aldosteronism, renal artery stenosis). They are often displayed as associated factors in the same patient [1].

Posterior reversible encephalopathy syndrome (PRES) is a clinical radiographic syndrome that has been described with hypertensive encephalopathy, eclampsia, and the use of cytotoxic drugs. In acute, severe hypertension (HTN), PRES results from an acute elevation of BP beyond the upper limits of cerebral autoregulation. The percent of elevation and the severity of BP over baseline are important with approximately 75% of patients have moderate to severe hypertension at presentation [2].

Here, we will describe SLE-patient with long-term RHTN, who presents with PRES and will discuss a rare and hard to diagnose the underlying cause of RHTN, which is finally diagnosed as carbamazepine induced HTN with response to discontinuation of the offending agent. This case report examines one such presentation in line with the SCARE guidelines [3].

## 2 Presentation of case

A 26- year old female was admitted to our emergency department due to tonic-clonic seizures followed by confusion and visual disturbance, in addition to fever and diarrhea. The past medical history consisted of systemic lupus erythematosus (SLE) at age of 16y, which was diagnosed after oral ulcers, malar rash, cerebrovascular accident, and seizures. A year after, the patient developed lupus nephritis (LN) type IV and received six courses of cyclophosphamide (CYP) pulse therapy with prednisone, then switched to mycophenolate mofetil (MMF). She had end-stage renal disease (ESRD) for the past year with two sessions of hemodialysis (HD) per week, pulmonary embolism, thrombosis in the right upper limb, and frequent pulmonary effusions. Also, the family described a long-standing uncontrolled and RHTN from the beginning of SLE.

Her medications included: prednisolone 10mg/d, carbamazepine 200mg/d, atorvastatin 10mg/d, rivaroxaban 2.5mg/d, erythropoietin (EPO) after HD sessions, methyldopa 250mg/bid, carvedilol 3.125mg/bid, and a combination of amlodipine/valsartan/hydrochlorothiazide (5/160/12.5)/bid.

Physical examination on emergency, BP 210/140 mmHg, pulse 97/min, and grade II edema in lower limbs. After urgent administration of diazepam and intravenous (IV) labetalol, she returned consciously and mildly confused with BP 190–200/120-140 mmHg. Neurological exam was as follows: Glasgow Coma Scale (GCS) 14, hyperreflexia, pupils reactive to light was normal, Babinski sign was negative, muscle strength and power were normal, visual blindness with sparing of abilities to perceive light and moving (cortical blindness). Laboratory tests on admission are shown in Table 1. Computed tomography (CT) scan of the brain showed an old infarction and ophthalmoscopy showed papilledema grade III.

**Table 1:** Laboratories on admission.

WBC	5.5	Na	137	CSF analysis	
HB	7.4	K	3.7	WBC	8
HT	21.7	Cl	99	RBC	100
PLT	141	Ca	8.6	Glu	53
Ur	154	P	4.2	Protein	256
Cr	5.2	CRP*	8.4	LDH	64
Glu	105	ESR	10	S. LDH	323
TP	5.7	PH	7.42	S.Glu	114
ALB	3.4	HCO3	18.8	PCR HSV	Neg
AST	23	C3^	58		
ALT	13	C4ᵒ	22		

WBC; white blood count, HB; hemoglobin, HT; hematocrit, PLT; platelets, Ur; urea, Cr; creatinine, Glu; glucose, TP; total protein, ALB; albumin, AST; Aspartate transaminase, ALT; alanine aminotransferase, Na; sodium, K; potassium, Ca; calcium, P; phosphorus, CRP*; C-Reactive Protein (up to 6 mg/dl), ESR; erythrocyte sedimentation rate, HCO3; bicarbonate, C3; complement C3 (range 90–180), C4; complement C4 (range 10–40), CSF; cerebrospinal fluid, RBC; red blood cells, PCR HSV; herpes simplex virus.

**Fig. 1:**
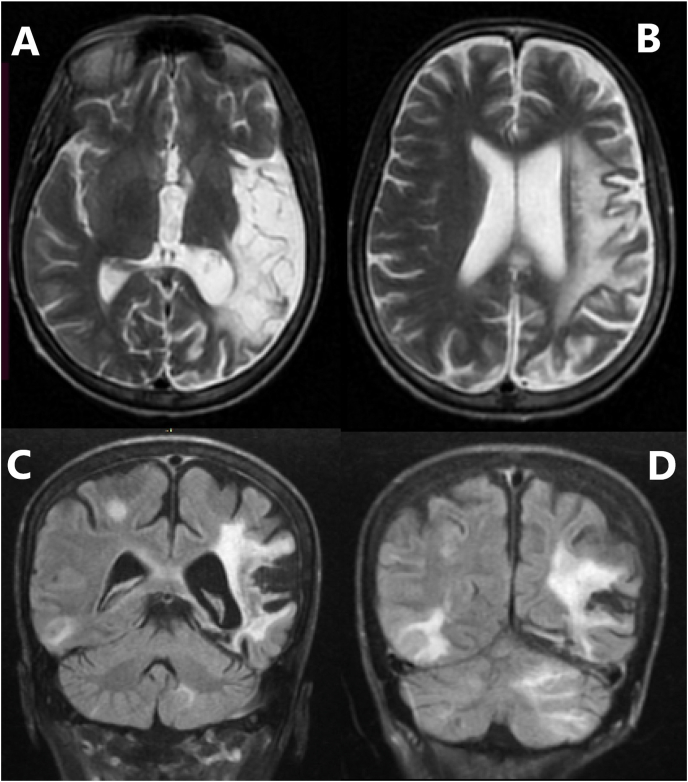
T2 weighted MRI: A; Axial T2 shows hyperintense signal in the left paretial and temporal lobes indicates an old infarction. B; Axial T2 shows hyperintense signal in the left paretial, temporal, occipital lobes C; Coronal T2 shows hyperintense signal in the left paretial and temporal lobes, atrophy of gray matter of the partial lobe shows as hypointense signal (arrow), tow foci of hyperintense-white matter lesions in paretial and temporal lobes. D; Coronal T2 shows hyperintense signal in the left paretial and temporal lobes, hyperintense-white matter lesion in right temporal lobe, hyperintense signal in left cerebellum.

**Fig. 2:**
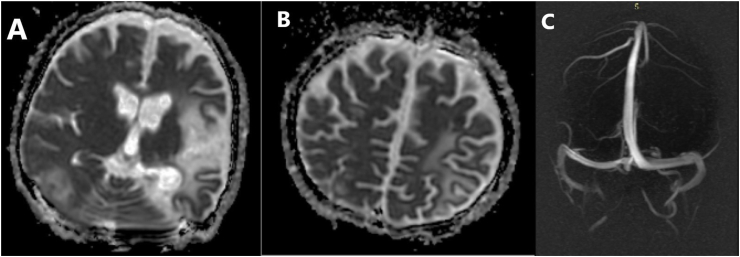
ADC and MRV: A; Axial ADC shows hyperintense signal in the left paretial and occipital lobes and hyperintense signal in the right occipital lobe. B; Axial ADC shows hyperintense signal in the left paretial and occipital lobes. C; Normal MRV with no venous thrombosis.

On day 1–2 of admission; although the patient received a continuous infusion of labetalol along with an increase of antihypertensive doses, BP slightly decreased to 160–180/110–130 mmHg (Table 2) but the confusion, hyperreflexia and cortical blindness were resolved. On day 3, MRI showed a previous infarction with novel findings of white matter edema compatible with PRES (Fig. 1, Fig. 2). HD session with fluid removal was applied and IV labetalol was discontinued due to the reverse of neurologic and visual abnormalities (Table 2). On the following days, however, antihypertensive drugs were increased to maximum doses with the second HD session, the patient still had high measures of BP (Table 2).

**Table 2:** Blood pressure monitoring and antihypertensive drugs modifications.

Day of admission	BP range	HD^a^, ^b^	Drugs and doses^b^
Admission	210/140	–	carvedilol 3.125mg/bid, amlodipine/valsartan/HCT *(5/160/12.5)/bid, methyldopa* 250mg*/bid*
D 1	170-180/120-140	–	*IV labetalol*^c^, carvedilol 3.125mg/bid, amlodipine/valsartan/*HCT (5/160/12.5)/bid,* methyldopa 250mg/tid*,* furosemide 20mg/IV/q6h
D 2	160-180/110-130	–	*IV labetalol*^c^, carvedilol 3.125mg/bid, amlodipine/valsartan/*HCT (5/160/12.5)/bid,* methyldopa 500mg/tid, furosemide 40mg/IV/q6h
D 3	160-180/110-140 MRI	Session	*IV bolus labetalol*^d^*, carvedilol 6.*25 mg*/bid*, amlodipine/valsartan/*HCT (5/160/12.5)/bid,* methyldopa 750mg/tid*, furosemide* 40mg*/IV/q6h*
D 4-5	170-200/120-140	–	IV bolus labetalol^d^, carvedilol 6.25 mg/bid, amlodipine/valsartan/*HCT (5/160/12.5)/bid,* methyldopa 1000mg/tid, *furosemide* 40mg*/IV/q6h.*
D 7	170-180/120-130 No edema	Session	IV bolus labetalol^d^, carvedilol 6.25 mg/bid, amlodipine/valsartan/*HCT (5/160/12.5)/bid, methyldopa* 1000mg*/tid, furosemide* 40mg*/IV/q6h*, *diltiazem* 60mg*/once*.
D8	180-190/130	–	IV bolus labetalol^d^, carvedilol 6.25 mg/bid, amlodipine/valsartan/*HCT (5/160/12.5)/bid, methyldopa* 1000mg*/tid, furosemide* 40mg*/IV/q6h*, diltiazem 60mg/once.
D10	180-190\ 110-120 Carbamazepine discontinuation	–	IV bolus labetalol^a^, ^b^, ^c^, ^d^, carvedilol 6.25 mg/bid, amlodipine/valsartan/*HCT (5/160/12.5)/bid, methyldopa* 1000mg*/tid, furosemide* 40mg*/IV/q6h*, diltiazem 60mg/once.
D 11- 12 Discharged	170-180/120-130	–	carvedilol 6.25 mg/bid, amlodipine/valsartan/*HCT (5/160/12.5)/bid, methyldopa* 1000mg*/tid,* diltiazem 60mg/once.
D15	120-130/70-85 Before HD session	–	same drugs
After a week	110/75	–	same drugs and start to withdraw

Italicize words refers to drugs changing in this day.

a Hemodialysis with fluid remove.

b Curved line refers to change in drugs or dose depend to previous day.

c Continuous IV labetalol infusion.

d IV labetalol bolus as needed.

### 2.1 Solving the diagnostic challenge? DDX?

Several etiologies were implicated in RHTN in this patient such as ESRD, LN, anemia, SLE, volume overload due to inadequate HD sessions, dietary non-compliance and might be inadequate doses of antihypertensive drugs [1,4,5].

**Fig. 3:**
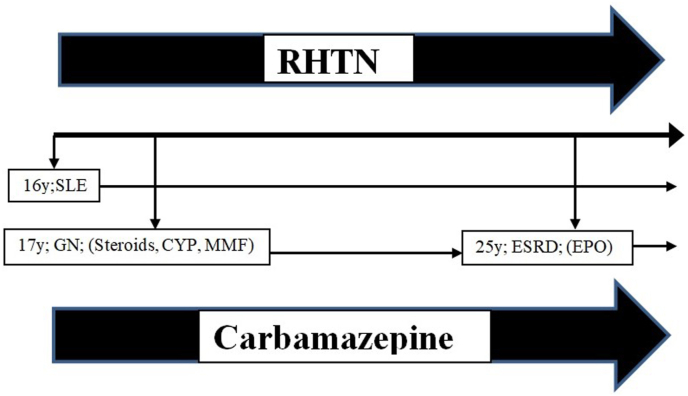
Timeline of the patients' status: SLE and carbamazepine are constant all along the course with RHTN. CYP; cyclophosphamide, MMF; mycophenolate mofetil, EPO; erythropoietin.

We review the patients’ status in more specific details and draw a timeline (Fig. 3). The family described RHTN from the beginning of SLE-diagnosis, before LN -which progress to ESRD in the past year- and despite dietary restrictions, intensifying of HD sessions and maximum tolerated doses of antihypertensive agents in the following years. Also, anemia was excluded with transfusion of blood unit.

Based on all previous, this grows a suspicion of the possibility of SLE being a cause of RHTN but still needs to exclude drugs effects. Depending on patient history, EPO was started in the last year, and steroids were discontinued several times in the past years, in the context of SLE treatment, but still suffered from uncontrolled HTN. As shown in the timeline (Fig. 3), the only constant drug from the beginning is carbamazepine. By reviewing the literature, we found that, in rare cases, carbamazepine was reported as a cause of uncontrolled HTN, so we discontinued carbamazepine and converted the patient to levetiracetam on day 10 (Table 2).

The family was discharged on their responsibility on day 12 and was given recommendations for drugs, BP monitoring, and dietary restrictions. Three days later, on day 15 and before the scheduled HD session, BP returned to normal values (120–130/70-85 mmHg) with the same prescribing drugs. A week after, we started to withdraw doses (Table 2) and the patient still had normal BP masseurs after two months of follow-up.

The clinical course in this patient suggests a long-standing RHTN- induced by carbamazepine due to reducing levels of antihypertensive drugs. On this occasion, diarrhea caused aggravation of drugs reduction that causes emergent HTN, which leads to PRES.

## 3 Discussion

To the best of our knowledge, carbamazepine-induced HTN was described in fourteen cases [[6], [7], [8], [9], [10], [11], [12], [13], [14], [15], [16], [17], [18], [19]]. Over the past ten years, a literature review yielded seven patients of carbamazepine-induced HTN (Table 3) [[9], [10], [11], [12], [13], [14], [15]]. Five patients had a history of HTN [9,11,[13], [14], [15]], two patients developed de novo HTN with carbamazepine initiation [10,12], and the highest systolic blood pressure reached 290 mmHg [12]. Four patients were received carbamazepine for treat trigeminal neuralgia [[10], [11], [12],^14^]. All cases described RHTN, that resolved with discontinuation of carbamazepine, one of these cases reported the administration of one intravenous with other five oral of antihypertensive drugs [13].

**Table 3:** Literature review of carbamazepine-induced hypertension.

Author	Age and medical history	Carbamazepine dose^a^, ^b^	BP^b^ mmHg	Antihypertensive drugs
(9)Ryul Kim et al. 2020	81y HTN occipital neuralgia	200 mg/bid	213/85 Developed PRES	candesartan 4mg, bisoprolol 1.25 mg, torsemide 5 mg
(10)Furuta N et al., 2009	21y trigeminal neuralgia	400 mg/d	170/126 Developed PRES	Nicardipine, amlodipine, valsartan
(11)Aamer Ubaid et al., 2019	45y HTN trigeminal neuralgia	300 mg/d	210/100	lisinopril 20 mg, HCT 25 mg
(12)Preeti Kharb et al., 2015	74y trigeminal neuralgia	300 mg/d	290/110	Telmisartan/HCT 40/12.5 mg
(13)Seon-Jae Kim et al., 2013	73y HTN syringomyelia	150mg/tid	215-200/104-95	Amlodipine 5mg/bid, perindopril 8mg/bid, nifedipine 30mg/bid, HCT 12.5 mg, arotinolol hydrochloride 5mg/bid, IV perdipine 5mg
**(14)Zylfije Hundozi et al** 2016	70 y HTN trigeminal neuralgia	800mg/d	180/110	Lizinopril 20 mg, HCT 25 mg
(15)Y.Akamine et al. 2015	53y HTN Schizophrenia	600 mg/d	160/103	Amlodipine 5mg

HTN; hypertension, y; year, bid; twice daily, tid; three times daily, d; day, HCT; *hydrochlorothiazide, IV; intravenous.*

a Highest dose of carbamazepine.

b The highest measure of blood pressure.

In our patient, several etiologies might explain RHTN, as mentioned before, however, a long-term complicated SLE is the most likely offending cause. The association between RHTN and SLE is obvious with the incidence rate is almost two-fold in patients with SLE compared to controls (10.1 versus 6.2 cases per 1000) [2]. This might cause a misdiagnosis of RHTN as a consequence of SLE, in this patient, without excluding other rare etiologies.

PRES is often presented with seizures, usually tonic-clonic, and neuroimaging is considered an essential tool of diagnosis, which usually shows a symmetrical white matter edema particularly in parieto-occipital regions. Although PRES is described in emergent HTN alone, it appears to be more common with comorbid conditions such as SLE. Also, patients will often improve dramatically with BP lowering [2]. Only three patients developed neurologic deficit in the context of severe HTN induced by carbamazepine, which was represented by PRES in two patients, and the third patient had transient neurologic symptoms [[8], [9], [10]]. In the current case; PRES is triggered by emergent HTN, with asymmetrical white matter lesions on MRI. However, all symptoms were retarded with BP lowering, carbamazepine caused resistance to almost all hypertensive classes, eight hypertensive drugs with maximum doses were used.

Various mechanisms were described to explain carbamazepine-induced HTN as follow: the most common theory is reduced levels of antihypertensive drugs as a result of the enhancement of CYP3A, inducing of P-glycoprotein (P-gp) transporter, altered antidiuretic hormone and changes in central noradrenergic mechanism [7,8,15]. Other study suggested similar structural and pharmacologic properties of carbamazepine with tricyclic antidepressants. These agents induce orthostatic hypotension and reflex tachycardia, which may be attributable to alpha adrenergic-blockade in the peripheral vasculature. Also, HTN was demonstrated in both drugs [7,20].

Carbamazepine seems not dose-dependent since it was associated with HTN even at very low doses (50 mg) and low therapeutic levels (0.4 μg/mL) [8,14]. Moreover, multiple classes of antihypertensive drugs were used in our patient, in addition to de novo HTN reported in previous reports [10,12]. These points make the activation of CYP450 enzymes seem not the only implicated mechanism and suggest multiple mechanisms for carbamazepine induce HTN. However, in our case, RHTN is most likely due to chronic reduction of antihypertensive levels, that aggravated by diarrhea in this occasion.

The elimination half-life of carbamazepine about 12–17 hours in adults, 72% urine excretion and the data of elimination by hemodialysis is controversial [[21], [22], [23]]. So, in this HD patient, these points gave the idea for awaiting a few days for carbamazepine elimination before judging its effects on HTN.

In the end, determining the secondary etiology of RHTN, in this patient, is considered a diagnosis of a challenge due to the coincidence of ESRD with SLE and the rarity of this side effect of carbamazepine.

## 4 Conclusion

Here we described SLE-patient with long-standing uncontrolled HTN, who presented with PRES syndrome. Conversion of carbamazepine to levetiracetam yielded in the resolve of RHTN. Side effects of drugs, even if rare, should always exclude in the approach of RHTN especially in ESRD-patients with multiple comorbidities. This case report is not sufficient to recommend BP monitoring after carbamazepine prescription but it is sufficient to warn clinicians of this side effect. Furthermore, future prospective studies, in patients using carbamazepine, should be applied to estimate the real proportion of HTN- induced by carbamazepine and to define its mechanisms in HTN.

## Sources of funding

This research did not receive any specific grant from funding agencies in the public, commercial, or not-for-profit sectors.

## Ethical approval

Written informed consent was obtained from the patient for publication of this case report and accompanying images, in line with local ethical approval requirements and in accordance with the helsinki declaration.

## Sources of funding

This research did not receive any specific grant from funding agencies in the public, commercial, or not-for-profit sectors.

## Author contribution

Mohammad Alsultan writes the manuscript, literature search, treat and follow up the patient and submitted the article. Kassem Basha made article corrections, literature search, and supervised the case.

## Consent

Written informed consent was obtained from the patient for publication of this case report and accompanying images. A copy of the written consent is available for review by the Editor-in-Chief of this journal on request.

## Registration of research studies

1. Name of the registry: N\A
2. Unique Identifying number or registration ID: N\A
3. Hyperlink to your specific registration (must be publicly accessible and will be checked): N\A

## Guarantor

The corresponding author is the guarantor of this manuscript.

## Provenance and peer review

Not commissioned, externally peer-reviewed.

## Declaration of competing interest

The author declares that they have no conflicts of interest regarding this study.

The author declares that it has not been published elsewhere and that it has not been submitted simultaneously for publication elsewhere.
